# Autoantibodies against the replication protein A complex in systemic lupus erythematosus and other autoimmune diseases

**DOI:** 10.1186/ar2000

**Published:** 2006-07-17

**Authors:** Yoshioki Yamasaki, Sonali Narain, Liza Hernandez, Tolga Barker, Keigo Ikeda, Mark S Segal, Hanno B Richards, Edward KL Chan, Westley H Reeves, Minoru Satoh

**Affiliations:** 1 Division of Rheumatology and Clinical Immunology, Department of Medicine, University of Florida, PO Box 100221, Gainesville, Florida, 32610, USA; 2 Department of Pathology, Immunology, and Laboratory Medicine, University of Florida, PO Box 100221, Gainesville, Florida, 32610, USA; 3 Department of Oral Biology, University of Florida, PO Box 100424, Gainesville, Florida, 32610, USA; 4 Division of Nephrology, Department of Medicine, University of Florida, PO Box 100221, Gainesville, Florida, 32610, USA

## Abstract

Replication protein A (RPA), a heterotrimer with subunits of molecular masses 70, 32, and 14 kDa, is a single-stranded-DNA-binding factor involved in DNA replication, repair, and recombination. There have been only three reported cases of anti-RPA in systemic lupus erythematosus (SLE) and Sjögren syndrome (SjS). This study sought to clarify the clinical significance of autoantibodies against RPA. Sera from 1,119 patients enrolled during the period 2000 to 2005 were screened by immunoprecipitation (IP) of ^35^S-labeled K562 cell extract. Antigen-capture ELISA with anti-RPA32 mAb, immunofluorescent antinuclear antibodies (ANA) and western blot analysis with purified RPA were also performed. Our results show that nine sera immunoprecipitated the RPA70–RPA32–RPA14 complex and all were strongly positive by ELISA (titers 1:62,500 to 1:312,500). No additional sera were positive by ELISA and subsequently confirmed by IP or western blotting. All sera showed fine speckled/homogeneous nuclear staining. Anti-RPA was found in 1.4% (4/276) of SLE and 2.5% (1/40) of SjS sera, but not in rheumatoid arthritis (0/35), systemic sclerosis (0/47), or polymyositis/dermatomyositis (0/43). Eight of nine patients were female and there was no racial predilection. Other positive patients had interstitial lung disease, autoimmune thyroiditis/hepatitis C virus/pernicious anemia, or an unknown diagnosis. Autoantibody specificities found in up to 40% of SLE and other diseases, such as anti-nRNP, anti-Sm, anti-Ro, and anti-La, were unusual in anti-RPA-positive sera. Only one of nine had anti-Ro, and zero of nine had anti-nRNP, anti-Sm, anti-La, or anti-ribosomal P antibodies. In summary, high titers of anti-RPA antibodies were found in nine patients (1.4% of SLE and other diseases). Other autoantibodies found in SLE were rare in this subset, suggesting that patients with anti-RPA may form a unique clinical and immunological subset.

## Introduction

Autoantibodies in systemic autoimmune rheumatic diseases such as systemic lupus erythematosus (SLE) often recognize molecules involved in the critical biological functions of cells such as DNA replication, repair, and recombination, splicing, transcription, translation, and cell cycle control [1]. These target antigens are subcellular particles consisting of multiproteins often with DNA or RNAs. Furthermore, many of these autoantibodies are specific for particular diagnoses and have been used as a disease marker [1]. Some of these are also associated with certain clinical symptoms or subset of disease and are useful in monitoring certain organ involvement and predicting outcome.

Among molecules involved in DNA replication, PCNA (proliferating-cell nuclear antigen) was identified as a target of autoantibodies in SLE more than 20 years ago [2,3]. Later the PCNA was identified as an auxiliary protein of DNA polymerase delta [4]. Anti-PCNA is considered an SLE-specific serological marker along with anti-Sm, anti-ribosomal P, and anti-dsDNA, although its frequency in SLE is only about 2% [1,5]. PCNA is a part of the large complex replication machinery, but little is known about the autoimmune response in rheumatic diseases to other components involved in DNA replication. Replication protein A (RPA), a heterotrimer with subunits of molecular masses 70, 32, and 14 kDa (RPA70, RPA32, and RPA14, respectively), is a single-stranded DNA-binding protein with multiple and essential roles in almost every aspect of DNA metabolism, including replication, repair, and recombination [6]. Autoantibodies against RPA in rheumatic diseases have been described in only three cases of SLE and Sjögren syndrome (SjS) from a screening of about 150 sera [7,8]. No systematic analysis in the rheumatic diseases or clinical significance of this specificity in human SLE is available. The screening in the previous studies was only by western blot analysis with recombinant RPA70 and RPA32 [7,8]. The reactivity with native RPA has not been evaluated. Autoimmune B-cell epitopes are often discontinuous [9,10], recognize native conformational epitopes, and in some cases are poorly reactive in western blot [11,12]. There are also antibodies that recognize quaternary structure consisting of several protein components in snRNPs [13] and DNA-dependent protein kinase (DNA-PK) [14]. On the basis of these observations in other autoantibody systems, we suspected that the frequency of anti-RPA might have been underestimated as a result of their preferential recognition of the native molecule and because anti-RPA may be associated with a specific clinical subset of SLE. We performed systematic screening of autoantibodies against the native form of RPA using immunoprecipitation (IP) and antigen-capture ELISA in sera from patients with rheumatic diseases, and analyzed the clinical significance of these autoantibodies.

## Materials and methods

### Patients

A total of 1,119 subjects enrolled at the University of Florida Center for Autoimmune Diseases (UFCAD) in the period 2000 to 2005 were studied. The subjects included 276 patients with SLE, 43 with polymyositis/dermatomyositis (PM/DM), 47 with scleroderma (systemic sclerosis (SSc)), 35 with rheumatoid arthritis (RA), and 40 with SjS. Diagnosis was established by American College of Rheumatology criteria (SLE, SSc, and RA) [15-17], Bohan's criteria (PM/DM) [18], or the European criteria (SjS) [19]. Clinical information was from the UFCAD database. The protocol was approved by the University of Florida's Institutional Review Board. This study meets and is in compliance with all ethical standards in medicine, and written informed consent was obtained from all patients in accordance with the Declaration of Helsinki.

### Monoclonal antibodies against RPA

mAbs against RPA70 (clone 2H10) and RPA32 (clone 9H8), obtained by immunization of RPA from HeLa cells, were from Lab Vision Corp (Fremont, CA, USA). Other mAbs against RPA32 and RPA14, made by immunization of His_6_-tagged recombinant human protein, were from QED Bioscience Inc (San Diego, CA, USA).

### Immunoprecipitation and confirmation of anti-RPA

The proteins recognized by human sera were evaluated by IP of radiolabeled K562 cell extracts and SDS-PAGE as described [12]. In brief, cells were labeled with [^35^S]methionine and cysteine, lysed in 0.5 M NaCl, 2 mM EDTA, 50 mM Tris pH 7.5, 0.3% Nonidet P40 (0.5 M NaCl NET/Nonidet P40) buffer containing 1 mM phenylmethyl sulfonyl fluoride and 0.3 TIU (trypsin inhibitor units)/ml aprotinin, and immunoprecipitated with Protein A–Sepharose beads coated with 8 μl of human serum. Immunoprecipitates were washed three times with 0.5 M NaCl NET/Nonidet P40 and once with NET/Nonidet P40 followed by SDS-PAGE and autoradiography. The specificity for anti-RPA was confirmed on the basis of IP of the characteristic set of three proteins of 70, 32, and 14 kDa that co-migrated with the proteins immunoprecipitated by monoclonal antibodies against RPA. The positive reaction was also confirmed by strong reactivity in antigen-capture ELISA.

### Antigen-capture ELISA for anti-RPA

Antigen-capture ELISA for anti-RPA was performed as described previously for anti-Ku, anti-nRNP/Sm, anti-Su, and anti-RNA helicase A, with some modification [20]. In brief, microtiter plates were coated overnight with 2 μg/ml mAb against RPA70, RPA32, or RPA14 in 0.1 M Na_2_HPO_4_/NaH_2_PO_4_, pH 9.0 at 4°C. Plates were washed, blocked with 0.5% BSA NET/Nonidet P40 (50 mM Tris-HCl, pH 7.5, 150 mM NaCl, 2 mM EDTA, 0.3% Nonidet P40) for one hour at room temperature. K562 cells (10^8^) were sonicated twice in 2.5 ml of 0.5 M NaCl NET/Nonidet P40 for 45 seconds and the cell extracts were cleared by microcentrifugation at 12,000 r.p.m. 11,269 × g for 30 minutes at 4°C. Supernatants were harvested and the plates were incubated with 50 μl of cell extract. Wells on half of each of the plates were incubated with 0.5% BSA in 0.5 M NaCl NET/Nonidet P40 (50 μl per well) as control. After incubation for 1 hour, plates were washed three times with TBS Tween 20 (20 mM Tris-HCl, pH 7.5, 150 mM NaCl, 0.1% Tween 20), and incubated with 1:2,500 diluted human sera in 0.5% BSA in 0.5 M NaCl NET/Nonidet P40 at room temperature for 1 hour. After being washed, the plates were incubated for 1 hour with alkaline phosphatase-labeled mouse anti-human IgG (dilution 1:1,000; Sigma, St Louis, MO, USA) and developed; *A*_405 _was then measured. The absorbances of wells without K562 cell extracts were subtracted from those of wells containing cell extracts and were converted into units as described [21]. In some experiments, cell extracts made with 0.15 M NaCl NET/Nonidet P40 and with 0.5 M NaCl NET/Nonidet P40 buffer were compared.

In other experiments, inhibition of anti-RPA antibodies binding to RPA was examined. RPA was affinity purified on a microtiter plate as described above with the use of anti-RPA70 mAb. After washing, wells were incubated with singlestranded DNA (ssDNA; boiled and chilled calf-thymus DNA; Sigma), double-stranded DNA (dsDNA; S1 nuclease-treated calf thymus DNA; Sigma), or synthesized double-stranded RNA (dsRNA; polyinosinic acid cytidylic acid, poly I:C; Sigma) in TE buffer (10 mM Tris-HCl, pH 8, 2 mM EDTA) at 0.01 to 100 μg/ml, or with buffer alone, for 30 minutes. Wells were then incubated with 1:2,000 diluted anti-RPA-positive sera for 1 hour, followed by incubation with ALP mouse anti-human IgG mAb (1:2,000 dilution) for 1 hour and then developed.

### Anti-ssDNA and dsDNA ELISA

A microtiter plate was coated with ssDNA or dsDNA with the use of Reacti-Bind DNA coating solution (Pierce, Rockford, IL, USA) in accordance with the manufacturer's instructions. Calf thymus DNA (Sigma) boiled for 10 minutes and rapidly chilled on ice for 10 minutes, was used as ssDNA. dsDNA was made by digesting calf thymus DNA with S1 nuclease [22]. Wells were coated with ssDNA or dsDNA (3 μg/ml, 100 μl per well) for 2 hours at 22°C, washed with TBS/Tween 20 and blocked with 0.5% BSA NET/Nonidet P40. Wells were then incubated with sera diluted 1:500 in the same buffer, followed by ALP mouse anti-human IgG mAb, and then washed and developed. Absorbances greater than the mean plus 3 standard deviations of 20 normal controls were considered positive.

### Immunofluorescent antinuclear antibodies

Immunofluorescent antinuclear antibodies (ANA) in the sera were tested at 1:160 dilution with the use of HEp2 cells and 1:200 diluted Alexa 488 goat anti-human IgG (H and L chain specific; Molecular Probes, Eugene, OR, USA) as described [23].

### Western blot analysis

RPA was affinity-purified from K562 cell extracts with the use of mAb against RPA70. Cell extracts from 3 × 10^8 ^cells in 0.5 M NaCl NET/Nonidet P40 were immunoprecipitated with 15 μg of mAb against RPA70. Purified proteins were fractionated by 12% SDS-PAGE and transferred to a nitrocellulose filter [12]. A strip of the filter 3 mm wide was probed with 1 μg/ml mouse mAb against RPA or human anti-RPA-positive or control serum at a dilution of 1:500. Blots were then incubated with 1:2,000-diluted horseradish peroxidase-labeled goat IgG anti-mouse IgG (γ-chain specific; Southern Biotechnology, (Birmingham, AL, USA) or goat IgG F(ab')_2 _anti-human IgG (γ-chain specific, Southern Biotechnology) and developed with SuperSignal West Pico Chemiluminescent Substrate (Pierce).

### Statistical analysis

All statistical analysis was performed with Prism 4.0c for Macintosh (GraphPad Software, Inc., San Diego, CA, USA). Fisher's exact test was used for analysis of association of anti-RPA with other specificities. A relationship between ELISA with anti-RPA70 versus anti-RPA32 mAb was analyzed by Spearman correlation. Anti-RPA ELISA between groups was compared by using Kruskal–Wallis with Dunn's multiple comparison test.

## Results

### Screening and identification of anti-RPA antibodies

**Figure 1:**
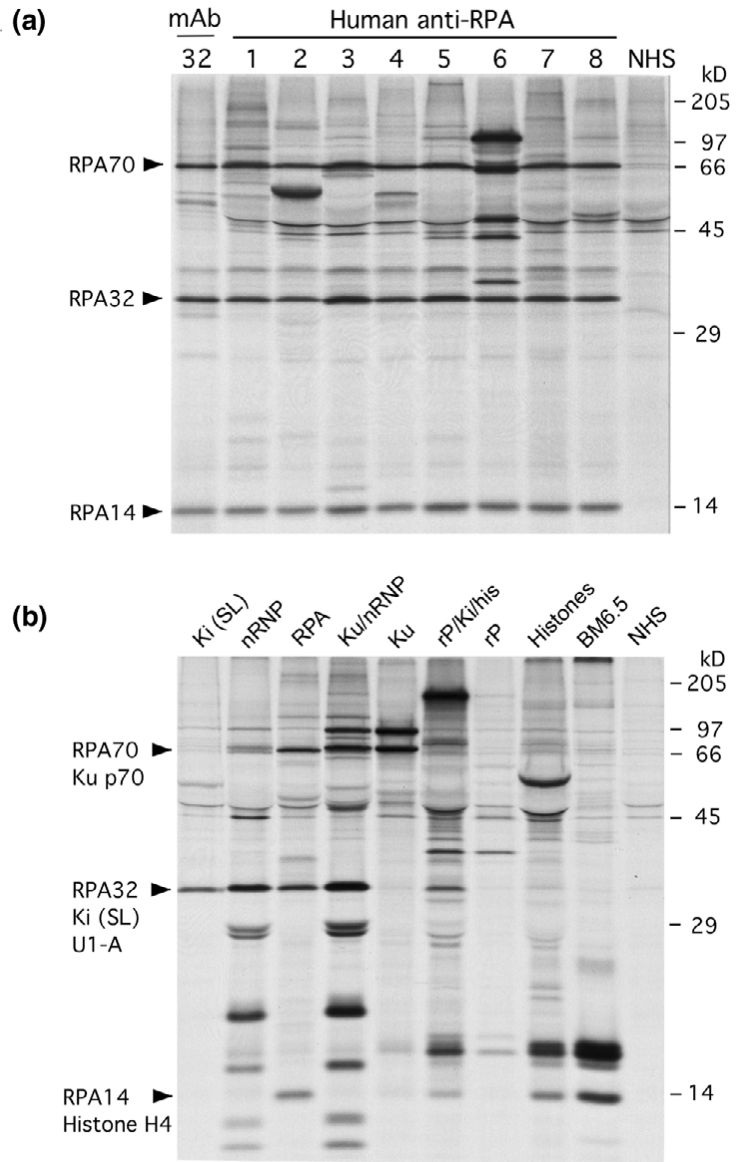
Immunoprecipitation of replication protein A (RPA). **(a) **Immunoprecipitation of RPA by mAbs and human autoimmune sera. ^35^S-labeled K562 cell extracts were immunoprecipitated with mAbs against RPA32 (lane 32), human sera with anti-RPA (lanes 1 to 4, SLE; lanes 5 to 8, others) or with normal human serum (NHS). Coexisting anti-Ro (lane 2) and anti-Su (lanes 3 and 8) are indicated by the open arrowheads. **(b) **Immunoprecipitation of lupus autoantigens that co-migrate or overlap with RPA. [^35^S]-labeled K562 cell extracts were immunoprecipitated with sera from patients with SLE (lanes labeled Ki to Histones except lane Ku) or PM (lane Ku), or mouse mAbs BM6.5 (anti-histones). These sera or mAbs recognize autoantigens co-migrate with components of RPA. RPA32 co-migrates with Ki (SL, lanes Ki and rP/Ki/his) and U1snRNP-A (U1-A, lanes nRNP and Ku/nRNP), RPA70 co-migrates with Ku p70 (lanes Ku/nRNP and Ku), and RPA14 co-migrates with histone H4 (lanes rP/Ki/his, Histones, and BM6.5). The specificities of human autoimmune sera are indicated. The numbers at the right are the molecular masses of protein standards. his, histones; rP, ribosomal P.

Autoantibodies against RPA were screened on the basis of the IP of the characteristic set of 70, 32, and 14 kDa proteins that co-migrated with those immunoprecipitated by anti-RPA32 mAb (Figure 1a, lane 32) from [^35^S]methionine-labeled K562 cell extracts. A representative IP by human anti-RPA sera and mAb against RPA32 is shown (Figure 1a). Nine human autoimmune sera (eight are shown in Figure 1a, lanes 1 to 8) clearly immunoprecipitated all three RPA proteins that co-migrated with those immunoprecipitated by mAb against RPA32 (Figure 1a, lane 32). During the screening, it was noted that each component of RPA co-migrated or overlapped with known lupus-related autoantigens on SDS-PAGE. Examples of lupus-related autoantigens that co-migrate with RPA are shown in Figure 1b. RPA32 co-migrates with the U1snRNPs-A protein (U1-A, immunoprecipitated by anti-nRNP and anti-Sm antibodies), that can be found in about 40% of SLE sera [5], and Ki (SL) antigen, which is recognized by about 10% of SLE sera [24,25]. RPA70 co-migrates with the p70 subunit of the Ku/DNA-PK antigen, which is recognized by about 6% of sera from SLE and other diseases [20]. RPA14 co-migrates with the histone H4 of the core histone complex [20,21] immunoprecipitated by certain autoimmune sera. If the molecular masses of proteins are not carefully compared, the pattern by anti-ribosomal P can also appear similar to that of RPA; in particular the coexistence of Ki or U1snRNPs in the same serum sample can be confusing. From the routine screening of autoantibodies by IP, nine sera were found to immunoprecipitate RPA; however, the co-migration of components of RPA with other lupus autoantigens shown in Figure 1b suggests that some anti-RPA sera might have been overlooked when other specificities coexisted. Thus, screening of sera by antigen-capture ELISA using mAbs to RPA was performed to find additional anti-RPA-positive sera that might have been overlooked by screening with IP.

mAbs established by immunization of recombinant histidine-tagged RPA32 or RPA14 proteins (QED Bioscience) did not efficiently immunoprecipitate RPA from K562 cell extracts (not shown) though they were positive by western blot (Figure 3b see below). In contrast, mAbs against RPA70 (clone 2H10) and RPA32 (clone 9H8) made by immunization of RPA from HeLa cells (Lab Vision) immunoprecipitated RPA from K562 cell extracts (Figure 1) and worked well in an antigen-capture ELISA after establishing appropriate conditions (see below). Anti-RPA32 mAb clearly immunoprecipitated all three components from K562 (Figure 1a, lane 32), HEp-2, and HeLa cells (not shown). Anti-RPA70 mAb efficiently immunoprecipitated all three components of RPA from HEp-2 cells but the IP of RPA32 and RPA14 from K562 and HeLa cells was very weak (not shown).

**Figure 2:**
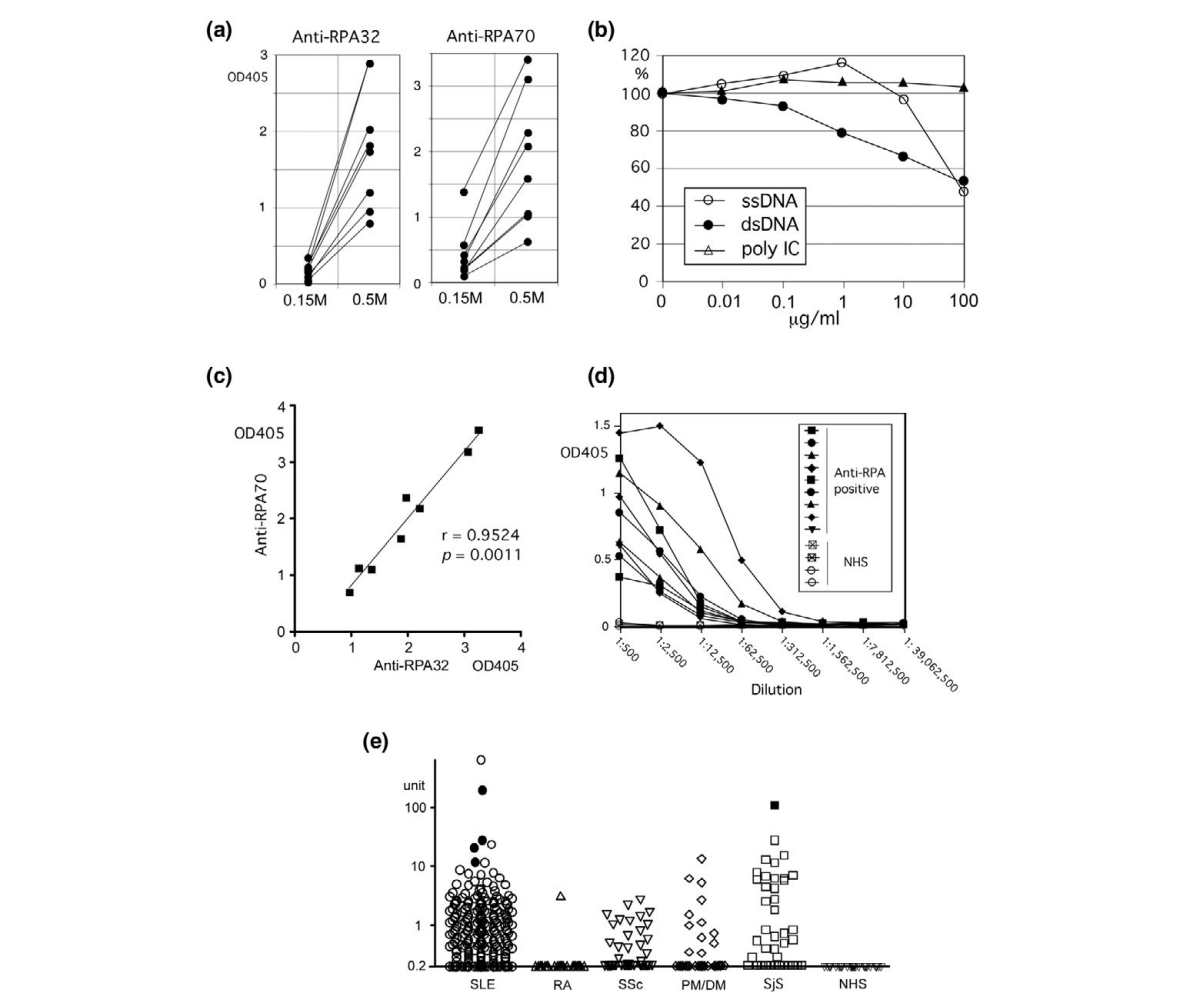
Anti-replication protein A (RPA) antigen-capture ELISA. **(a) **Effects of NaCl concentration of the cell extracts on the reactivity of anti-RPA human sera. ELISAs were performed as described in the Materials and methods section with mAbs against RPA32 or RPA70 to coat ELISA plates and to capture RPA from K562 cell extracts, which were prepared in buffer containing either 0.15 M or 0.5 M NaCl. Sera diluted to 1:500 in 0.5 M NaCl NET/Nonidet P40 were tested. **(b) **Effects of single-stranded DNA (ssDNA), double-stranded DNA (dsDNA), or double-stranded RNA (dsRNA) on the reactivity of human anti-RPA autoantibodies. Affinity-purified RPA on a microtiter plate was incubated for 30 minutes with ssDNA, dsDNA, or dsRNA (poly I:C) at concentrations of 0.01 to 100 μg/ml or with buffer alone. Wells were then incubated with 1:2,000 diluted anti-RPA-positive sera followed by ALP mouse mAb anti-human IgG, and developed. The percentage reactivity compared with RPA incubated with buffer alone (100%) is shown. ssDNA or dsDNA, but not dsRNA, inhibited the human anti-RPA binding in a dose-dependent manner. **(c) **Correlation between levels of anti-RPA by antigen-capture ELISA with mAbs against RPA32 and against RPA70. The reactivity of eight anti-RPA-positive human autoimmune sera in ELISA with mAbs against RPA32 and against RPA70 was compared. Spearman *r *= 0.9524, *p *= 0.0011. **(d) **Titration curves of anti-RPA-positive human sera. Titration curves of nine anti-RPA-positive autoimmune sera and four normal human sera (NHS) were created by ELISA with mAb against RPA32. K562 cell extracts in 0.5 M NaCl NET/Nonidet P40 buffer were used and sera were serially diluted 1:5 starting from 1:500. **(e) **Screening of anti-RPA antibodies in sera from patients with various systemic rheumatic diseases by ELISA. Sera from SLE (*n *= 276), rheumatoid arthritis (RA; *n *= 35), SSc (*n *= 47), PM/DM (*n *= 43), SjS (*n *= 40), and normal control (NHS, *n *= 30) were tested at 1:2,500 dilutions by ELISA with mAb against RPA32. SLE (*p *< 0.001 versus RA, *p *< 0.05 versus SSc, *p *< 0.01 versus PM/DM, *p *< 0.001 versus NHS) and SjS (*p *< 0.001 versus RA or NHS, *p *< 0.05 versus SSc, *p *< 0.01 versus PM/DM) showed high reactivity. RA versus SSc, *p *< 0.05; SSc versus NHS, *p *< 0.05; all other pairs were not significant. All comparisons were made with the Kruskal–Wallis test with Dunn's multiple comparison test. Open symbols, immunoprecipitation negative; filled symbols, immunoprecipitation positive. SjS, Sjögren syndrome; SSc, systemic sclerosis.

The reactivity of anti-RPA IP-positive sera in antigen-capture ELISA with the use of cell extracts made with 0.15 M NaCl and with 0.5 M NaCl in otherwise identical lysis buffer (50 mM Tris-HCl, 2 mM EDTA, 0.3% Nonidet P40) was compared (Figure 2a). All eight sera tested reacted weakly when cell extracts were made with 0.15 M NaCl buffer; however, the absorbance increased markedly when the 0.5 M NaCl cell extracts were used with either anti-RPA32 or anti-RPA70 mAb (Figure 2a). These results suggest that RPA can be extracted more efficiently in high NaCl, and/or that the dissociation of interacting proteins and DNA by high NaCl and possibly secondary conformational changes help the binding of anti-RPA antibodies in autoimmune sera.

We examined whether the binding of DNA to RPA can interfere the reactivity of anti-RPA autoantibodies by incubating affinity-purified RPA with ssDNA, dsDNA, or dsRNA before antibody binding. When the RPA was incubated with ssDNA or dsDNA before the reaction with autoimmune sera, the binding of all eight sera tested was inhibited by about 50% by ssDNA (47.2 ± 11.2% (mean ± SD), range 34.0 to 66.9%) or dsDNA (52.0 ± 11.8%, range 36.3 to 63.2%), but not by dsRNA (Figure 2b) at 100 μg/ml. dsDNA showed stronger inhibition than ssDNA in all eight cases, inhibiting anti-RPA binding in a dose-dependent manner up to 0.1 to 1 μg/ml. At 1 μg/ml, ssDNA inhibited by more than 10% in zero of eight cases, whereas dsDNA showed the same effects in seven of eight cases. These data are consistent with Figure 2a and suggest that the binding of DNA to RPA in 0.15 M NaCl was at least partly responsible for the poor binding of anti-RPA antibodies against RPA when cell extracts were made in 0.15 M NaCl buffer (Figure 2a).

Anti-ssDNA and anti-dsDNA antibodies were positive in four of nine and one of nine cases by ELISA, respectively (not shown). In cases with high anti-ssDNA antibodies, reactivity with RPA after incubation with ssDNA increased at a moderate concentration of ssDNA. This is consistent with the reactivity of anti-ssDNA antibodies against ssDNA that binds to RPA. In the presence of a high concentration of ssDNA, inhibitory effects on anti-RPA–RPA binding seemed to be dominant compared with enhanced reactivity via anti-ssDNA antibody binding to ssDNA on RPA. However, at a low concentration of ssDNA, inhibition on anti-RPA binding by ssDNA was minimal, whereas anti-ssDNA antibodies caused a false high binding via ssDNA on RPA.

When the reactivity of ELISA with anti-RPA70 mAb was compared with that of anti-RPA32 mAb, there was a nearly perfect correlation (Figure 2c; Spearman *r *= 0.9524, *p *= 0.0011). On the basis of these data, the screening of sera for anti-RPA antibodies was performed with anti-RPA32 mAb and cell extracts with 0.5 M NaCl NET/Nonidet P40. Considering the DNA-binding capability of RPA, sera were diluted in 0.5 M NaCl buffer to minimize the false-positive reactions caused by the binding of the DNA–anti-DNA immune complex to RPA.

Titration curves of the nine anti-RPA IP positive sera and four controls by ELISA with anti-RPA32 mAb are shown in Figure 2d. Sera were serially diluted 1:5, starting from 1:500 dilutions. All sera were clearly positive on ELISA and their titers were 1:12,500 in one case, 1:62,500 in six, and 1:312,500 in two, indicating that the titers of anti-RPA were as high as those of other high-affinity IgG autoantibodies in SLE.

**Figure 3:**
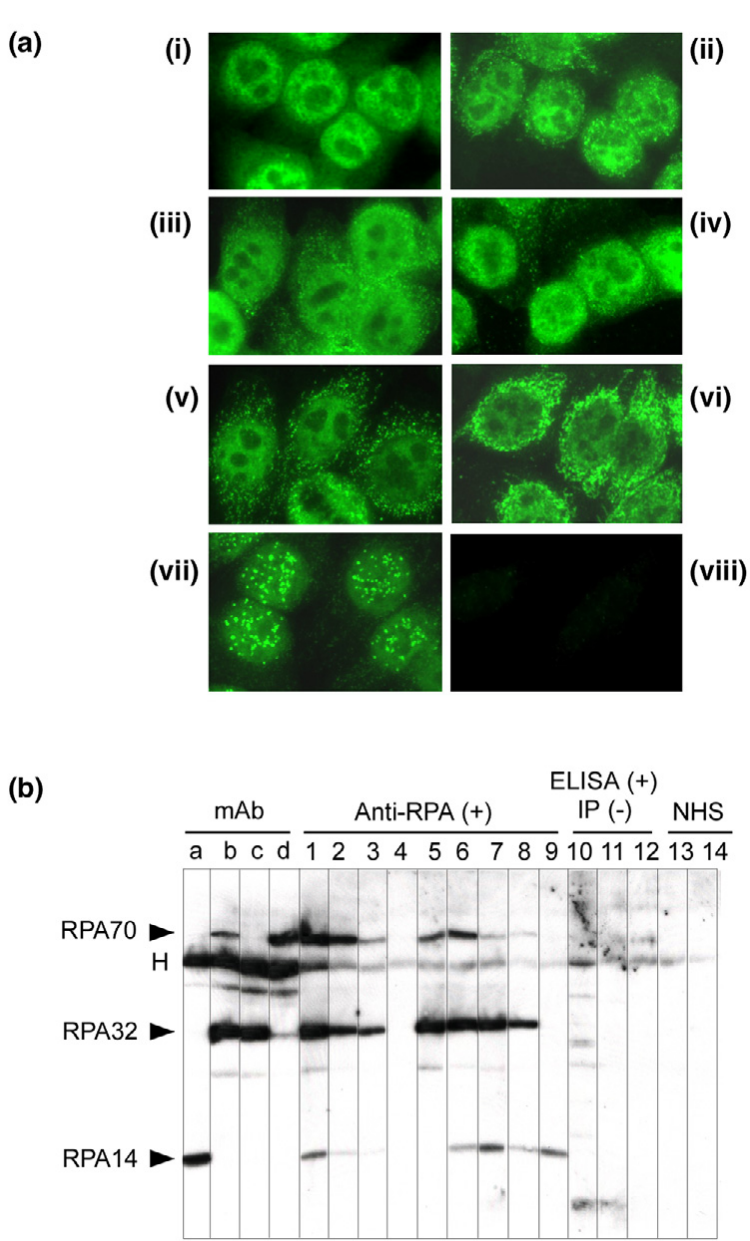
Immumofluorescent ANA and western blot with anti-RPA positive sera **(a) **Immunofluorescent ANA testing with anti-RPA-positive sera. HEp-2 cells were stained with mAb against RPA32 **(i)**, RPA70 **(ii)**, human autoimmune sera with anti-RPA (1:160 dilution, **iii–vii**), or normal control **(viii)**. All anti-RPA-positive sera showed nuclear fine speckled/homogeneous staining, similar to the staining by anti-RPA32 or anti-RPA70 mAb. Some sera had an additional immunofluorescent pattern from the other coexisting specificities; mitochondria **(vi) **and centromere **(vii)**. **(b) **Western blot analysis of anti-RPA antibodies. RPA was immunoprecipitated from K562 cell extract, fractionated by 12% SDS-PAGE, and transferred to a nitrocellulose filter. Strips of the filter were probed with mAbs against RPA (lanes a to d: a, RPA14; b, RPA32; c, RPA32; d, RPA70), anti-RPA immunoprecipitation-positive sera (lanes 1 to 9), anti-RPA ELISA-positive immunoprecipitation-negative sera (lanes 10 to 12), or control sera (normal human serum (NHS), lanes 13 and 14). H, mouse IgG heavy chain.

Sera from patients with SLE and other systemic rheumatic diseases were screened by antigen-capture ELISA (Figure 2e). As a group, sera from patients with SLE (*p *< 0.001 versus RA, *p *< 0.05 versus SSc, *p *< 0.01 versus PM/DM, *p *< 0.001 versus normal human serum (NHS)) and SjS (*p *< 0.001 versus RA or NHS, *p *< 0.05 versus SSc, *p *< 0.01 versus PM/DM) showed high reactivity. In addition to the five sera (four SLE, one SjS) that were confirmed for anti-RPA by IP (filled circles), there were sera that showed comparable or higher reactivity on ELISA. However, after careful evaluation of proteins immunoprecipitated by these sera and by western blotting, none of the additional ELISA positive sera were considered positive (Figure 3b, and data not shown). Thus, anti-RPA was found in nine cases by IP and no additional cases were found from ELISA screening. The false-positive reactivity of many SLE and SjS sera in this ELISA is probably due to their reactivity with DNA (see Figure 2a,b) and other proteins co-purified with RPA (see Figure 3b), similar to their false-positive reaction in anti-Ku ELISA [20]. Although all nine IP-positive sera showed high reactivity, the ELISA was not useful because of the poor signal:noise ratios and the high frequency of false positives.

### Immunofluorescent ANA test

All nine anti-RPA IP-positive sera showed a fine speckled/homogeneous nuclear staining (the staining of five cases is shown in Figure 3a, panels iii–vii) similar to that by anti-RPA32 mAb (Fig. 3a, panel i) or anti-RPA70 (Fig. 3a, panel ii). Some sera seem to have additional cytoplasmic staining, which is consistent with previous observations with affinity-purified anti-RPA antibodies [7]. One serum each among anti-RPA positive sera also had anti-mitochondria antibodies (Fig. 3a, panel vi) or anti-centromere antibodies (Fig. 3a, panel vii).

### Western blot analysis

Six of nine anti-RPA-positive sera reacted with all three components of RPA; of the remainder, one reacted with RPA70 and RPA32, one reacted with RPA14 only, and one was negative (Figure 3b, lanes 1 to 9; Table 1). Generally, the sera with higher levels of anti-RPA by ELISA showed strong reactivity in western blotting. Most sera reacted strongly with RPA32, followed by RPA70. Reactivity with RPA14 was generally weak. There was no relationship between reactivity with different components of RPA and diagnosis. Anti-RPA ELISA-positive IP-negative sera did not react with RPA but some reacted with other proteins co-purified by anti-RPA mAb (Figure 3b, lanes 10 to 12). These data explain the false positive results in ELISA given by some sera.

### Clinical and immunologic features of anti-RPA-positive patients

Nine patients with anti-RPA were identified out of total of 1,119 patients. Anti-RPA was found in 1.4% (4 of 276) in SLE (includes SLE-overlap syndrome) but not in other systemic autoimmune rheumatic diseases such as SSc (*n *= 47), PM/DM (*n *= 43), and RA (*n *= 35). However, anti-RPA was also found in five cases that do not fulfill SLE criteria including 1 of 40 (2.5%) with SjS. All except one case were female and there was no race predilection (Table 1). SLE criteria of the positive cases were not particularly characteristic except for that none of five cases (including one possible case that had three SLE criteria) had discoid rash or neurological symptoms (Table 2). One additional case possibly had SLE (leucopenia, lymphopenia, anti-dsDNA antibodies, ANA, and possible arthritis; included in Table 2). One case of each had SjS plus primary biliary cirrhosis, interstitial lung disease, autoimmune thyroiditis plus hepatitis C virus infection plus pernicious anemia, and one case without clinical information.

Frequency of coexisting other autoantibodies found in anti-RPA-positive versus anti-RPA-negative SLE patients was compared (Table 3). Interestingly, autoantibodies that can be found in up to 30 to 40% of SLE patients such as anti-snRNPs or anti-Ro were rare among anti-RPA-positive sera. None of the anti-RPA sera were positive for anti-nRNP, anti-Sm, and anti-La, and only one case was positive for anti-Ro (Figure 1a, lane 2, open arrowhead). Two cases had anti-Su (Figure 1a, lanes 3 and 8, open arrowhead). Anti-nRNP was significantly less common in anti-RPA-positive sera than in anti-RPA-negative SLE (*p *= 0.00122 by Fisher's exact test).

About 200 patients are enrolled to UFCAD every year. One case of anti-RPA was found in the year 2000, no cases in 2001 and 2002, four cases in 2003, three cases in 2004, and one case in 2005. It is possible that there is a year-to-year difference in the prevalence of anti-RPA (2001 to 2002 versus 2003 to 2004, *p *< 0.05 by Fisher's exact text) but the number is too small to be conclusive.

## Discussion

In the previous studies in rheumatic diseases, only three cases of SLE and SjS with anti-RPA have been described [7,8]. The first report described two cases with anti-RPA from the screening of 55 autoimmune sera by western blotting with RPA70 and RPA32 recombinant proteins [7]. One case was of SjS whose serum reacted with RPA70 and RPA32; the other was of SLE–SjS complicated with gastric lymphoma treated with radiotherapy [8], whose serum reacted with RPA32. A subsequent study from the same authors described 2 of 108 SLE sera that were positive in a western blot; these were a case reported previously and another case whose serum reacted with both RPA32 and RPA70 [8]. The frequency of anti-RPA in SLE was 1.9%, similar to that in the present study. Because certain autoantibodies preferentially recognize the native molecules, antibodies against native RPA were screened by IP in this study. Two sera (cases 4 and 9), which were negative for RPA70 and RPA32 in western blot, would have been missed if IP had not been used, although this was a relatively minor population (two of nine; 22% of anti-RPA-positive sera). ELISA may be helpful in identifyng additional anti-RPA-positive sera in theory; however, false positives via the reactivity of antibodies against DNA and proteins that interact with RPA seem to be quite common among patients with systemic rheumatic diseases, in particular in SLE and SjS (Figures 2b,e and 3b). The 'true' reactivity of anti-RPA antibodies was significantly inhibited by DNA (Figure 2b), consistent with the idea that autoantibodies recognize the functional site of the target molecule [1].

One study reported the frequent detection of anti-RPA autoantibodies in cancer patients [26]. HeLa cDNA expression library was screened with a high-titer ANA-positive serum from a breast cancer patient, and RPA32 was cloned. Sera from cancer patients were screened by ELISA with the recombinant RPA32 protein. Antibodies against RPA32 were found in 10.9% (87 of 801) in breast cancer patients and in 10.3% (4 of 39) in intraductal *in situ *carcinoma patients, in contrast with non-cancer controls (0 of 65) [26]. Various autoantibodies have been described in patients with cancer [27,28] and it is possible that anti-RPA is found in diseases other than systemic rheumatic diseases. However, because the previous study was based on ELISA alone, which is prone to false positives as shown in the present study, this finding will need to be verified in future studies with other methods. None of the anti-RPA-positive patients in this study had cancer.

In SSc and PM/DM, classifying patients into subsets based on their autoantibodies has been studied extensively [29,30]. Disease-associated autoantibodies rarely coexist in SSc and PM/DM. SSc-related autoantibodies against topoisomerase I, RNA polymerase I/III, fibrillarin, Th (7-2RNP), centromere, or PM-Scl seldom coexist and thus about 80% of SSc has one of these autoantibodies [30]. In PM/DM, patients with anti-aminoacyl tRNA synthetase antibodies have antibodies against only one of the synthetases, and other patients have anti-SRP, anti-PM-Scl, anti-Ku, and anti-nRNP [31]. Several autoantibodies including anti-Sm, anti-ribosomal P, anti-PCNA, and anti-dsDNA have been known to be specific for SLE [1,5]. However, in contrast with the finding in SSc or PM/DM, frequent coexistence of disease-specific autoantibodies has been reported in SLE [32]; many patients have more than one of anti-Sm, anti-dsDNA, and anti-ribosomal P. The present study suggests that anti-RPA-positive patients may form a unique group of SLE without other autoantibodies commonly found in SLE.

Many of the known autoantigens recognized by sera from patients with SLE are phosphoproteins including snRNPs, La, ribosomal P, DNA-PK, RNA polymerase II, histones, and nucleolin [5]. We have previously identified autoantibodies against RNA polymerase (RNAP) II in SLE sera that preferentially recognize the phosphorylated form of RNAP II but are unreactive with the unphosphorylated form of RNAP II [33]. In patients with SSc, autoantibodies specific for the phosphorylated form of RNAP II always coexisted with autoantibodies against another phosphoprotein, topoisomerase I, suggesting the role of phosphoamino acids in the autoimmune B-cell epitope [34]. In contrast, it has been shown that phophorylation is not necessary for ribosomal P antigens to be recognized by autoantibodies in SLE [35].

Both RNAP II and RPA are phosphorylated by exposure to ultraviolet [36,37] or chemicals such as hydroxyurea and camptothecin [38]. RPA has a role in sensing damaged DNA, and ultraviolet or certain chemicals induces the phosphorylation of RPA32 by DNA-PK, another target of autoimmune response in SLE, and cdc2 kinase [39]. The phosphorylated RPA32 becomes unstable, is dissociated from the RPA complex [40] and prevents RPA association with replication centers [41]. Although anti-RPA described in the present study recognizes the unphosphorylated form of RPA32, in contrast with the preferential recognition of the phosphorylated form of RNAP II by certain SLE sera [33], it is tempting to speculate that abnormal phosphorylation, disassembly of RPA, and degradation triggered by ultraviolet or chemicals are associated with the initiation of an autoimmune response to RPA. Whether anti-RPA is associated with photosensitivity or skin lesion in SLE, as described for anti-Ro antibodies [42], will be another point of interest that needs to be examined in future studies.

## Conclusion

High titers of anti-RPA antibodies were found in nine patients (1.4% of those with SLE and other diseases). Although anti-RPA seems to be a rare autoantibody specificity, it may represent a unique clinical and immunological subset of autoimmune disease that does not produce common lupus-related autoantibodies.

## Abbreviations

ANA = antinuclear antibodies; DNA-PK = DNA-dependent protein kinase; dsDNA = double-stranded DNA; dsRNA = double-stranded RNA; ELISA = enzyme-linked immunosorbent assay; IP = immunoprecipitation; mAb = monoclonal antibody; NHS = normal human serum; PCNA = proliferating-cell nuclear antigen; PM/DM = polymyositis/dermatomyositis; RA = rheumatoid arthritis; RNAP = RNA polymerase; RPA = replication protein A; SjS = Sjögren syndrome; SLE = systemic lupus erythematosus; snRNP = small nuclear ribonucleoprotein; SSc = systemic sclerosis; ssDNA = singlestranded DNA; UFCAD = University of Florida Center for Autoimmune Diseases.

## Competing interests

The authors declare that they have no competing interests.

## Authors' contributions

YY carried out the immunoassays, participated in the data analysis and in the design of the study, and drafted the manuscript. SN, LH, TB, KI, MSS, HBR, and WHR helped with data collection. TB also helped in editing the manuscript. EKLC provided technical help and advice for immunoassays, took immunofluorescent ANA pictures, and also helped edit the manuscript. MS designed and coordinated the study, performed the immunoassays and the data analysis, and also edited the manuscript. All authors read and approved the final manuscript.

## Figures and Tables

**Table 1:** Cases with anti-RPA autoantibodies

Case	Age	Sex	Race	Diagnosis	SLE criteria	ELISA (unit)	Western blot		
							
							RPA70	RPA32	RPA14
1	27	F	Black	SLE	7	189.0	++	++	+
2	45	F	Latin	SLE	5	11.5	++	++	+
3	35	F	Mixed	SLE	4	19.6	+	++	+
4	23	M	Black	SLE	6	27.0	-	-	-
5	41	F	White	Possible SLE	3	149.1	+	++	-
6	61	F	White	SjS, PBC	1	125	++	++	+
7	56	F	Black	ILD	1	> 625	+	++	+
8	48	F	White	Thyroiditis, HCV, pernicious anemia	1	81.8	+	++	+
9	51	F	Black	Unknown	1	34.4	-	-	+

SLE, systemic lupus erythematosus; SjS, Sjögren syndrome; PBC, primary biliary cirrhosis; ILD, interstitial lung disease; HCV, hepatitis C virus infection; ELISA, enzyme-linked immunosorbent assay; RPA, replication protein A.

**Table 2:** Systemic lupus erythematosus criteria of cases with anti-RPA

Criterion	Case 1	Case 2	Case 3	Case 4	Case 5
Malar rash	+	+	-	-	-
Discoid rash	-	-	-	-	-
Photosensitivity	+	Possible	-	-	-
Oral ulcers	+	+	+	-	-
Arthritis	+	+	-	+	Possible
Serositis	-	-	-	+	-
Renal disorder	+	Possible	-	+	-
Neurologic	-	-	-	-	-
Hematologic	-	-	+	+	+
Positive serology	+	+	+	+	+
Anti-Sm	-	-	-	-	-
Anti-dsDNA	+	+	+	+	-
Anti-phospholipid	+	-	+	-	+
Antinuclear antibodies	+	+	+	+	+
Number of criteria (out of 11 SLE diagnostic criteria)	7	5	4	6	3

dsDNA, double-stranded DNA; RPA, replication protein A.

**Table 3:** Frequency of autoantibodies (percentages) in patients with anti-RPA

Autoantibody	RPA(+), all (*n *= 9)	RPA(+), SLE (*n *= 4)	RPA(-), SLE (*n *= 272)
Anti-nRNP	0^a^	0	39^a^
Anti-Sm	0	0	15
Anti-Ro	11	25	33
Anti-La	0	0	11
Anti-Ku	0	0	2
Rib-P	0	0	8
Anti-RNA helicase A	0	0	6
Anti-Su	22	0	10

RPA, replication protein A; SLE, systemic lupus erythematosus; ^a^, *p *= 0.00122 by Fisher's exact test.
